# IL-7 is a Key Driver Cytokine in Spondyloarthritis?

**DOI:** 10.1155/2019/7453236

**Published:** 2019-05-29

**Authors:** Rafaela Silva Guimarães Gonçalves, Angela Luzia Branco Pinto Duarte

**Affiliations:** Hospital das Clínicas de Pernambuco, Brazil

## Abstract

The rationale for a type 17 signature in the pathogenesis of spondyloarthritis (SpA) has been increasing and being ratified in studies recently. IL-7 is a cytokine whose ability to stimulate IL-17 production in both innate and adaptive immunity cells has made it a promising target not only for a better understanding of the disease as well as an important potential therapeutic target in patients with SpA.

## 1. Introduction

**Figure 1:**
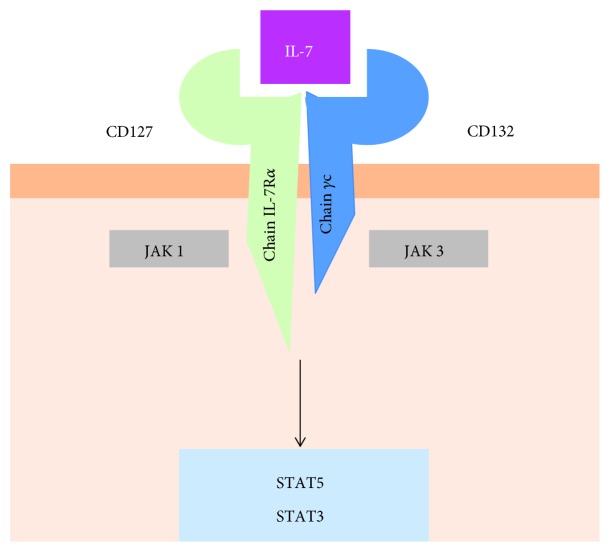
IL-7R*α* (CD127) associates with *γ*c to (CD132) form the IL-7R. The *γ*c cytokine signal via the Janus kinase- (JAK-) signal transducer and activator of the transcription (STAT3 or 5) pathway.

The human interleukin-7 gene (IL-7) is located on chromosome 8q12-13, and its molecular weight is 17.4 kDa [1]. It is classified as a cytokine of the hematopoietin family that includes IL-2, IL-3, IL-4, IL-5, IL-9, macrophage colony-stimulating factor (GM-CSF), IL-13, and IL-15 [2, 3]. This cytokine family shares the common receptor of the *γ* chain (*γ*c), also known as CD132. The IL-7 receptor (IL-7R) is a heterodimer (consisting of two subunits), interleukin-7-*α* (CD127) receptor, and *γ*c receptor (CD132) (Figure 1).

IL-7 already has a recognized function in B cell precursors and acts on both mature [4] and immature T cells [5–7], regulating homeostasis of the T cell population [8, 9], for example, IL-7 levels increase when T cell depletion is present for any reason [10–12]. The description of IL-7 receptor alpha chain mutations in patients with severe combined immunodeficiency (SCID) has confirmed that IL-7 is essential for the development of T cells [13].

The nonderived stromal bone marrow and epithelial cells are the main sources of IL-7 [10]. However, as showed by Ciccia et al. [14], Paneth cells also produce interleukin-7 in the intestine especially in patients with ankylosing spondylitis (AS). In addition, in activation by lipopolysaccharides in the intestine, hepatocytes can significantly increase IL-7 secretion [15]. After secretion, IL-7 binds to the extracellular matrix in lymphoid and nonlymphoid organs, including the skin, liver, and intestine [10].

Consistent with the above writing, IL-7 is a pleiotropic cytokine and plays a central role in the modulation of T and B cell development, in addition to T cell homeostasis. The potency and amplitude of the effects suggest that administration or neutralization of IL-7 may allow the modulation of immune function in patients with lymphocyte depletion or even in autoimmune diseases [2].

Since the most well-known function of IL-7 is that of shaping and regulating CD8 cytotoxic T cells, the interesting possibility of its role in diseases associated with the major class I histocompatibility complex, such as spondyloarthritis (SpA), arises. Several other roles in human and experimental arthritis have recently been defined [16, 17]: IL-7 stimulates the production of proinflammatory cytokines in experimental arthritis [18], influences ectopic lymphoid neogenesis [19], promotes osteoclastogenesis [20, 21], and abolishes the function of regulatory T cells (Treg) [22, 23].

SpA encompasses a group of chronic inflammatory conditions, which may involve the axial skeleton (sacroiliac spine and joints) and peripheral joints, as could be associated with extra-articular manifestations such as psoriasis, inflammatory bowel disease (IBD), and uveitis, and usually sharing a close association with HLA-B27 [24]. Theretofore, SpA was based on the premise of an imbalance, especially of adaptive immunity, encompassing the IL-23 axis as a polarization stimulator for a Th17 response, with consequent IL-17 and TNF productions [25, 26]. However, currently, the role of innate immunity cells as the main in the physiopathogenesis of SpAs has been growing exponentially [27, 28].

The idea that SpA is a disease mediated by inflammatory response type 17 (or type 3) has been growing, and therefore, IL-17 has a dominant role in the inflammatory and proliferative cascades of human SpA [29–31]. Rihl et al. showed high levels of mRNA and proteins IL-7 in peripheral SpA, which were even higher than in rheumatoid arthritis (RA) [32]. In addition, recent results indicate that the IL-7R pathway is locally unregulated in the colon of patients with severe IBD and may contribute to the maintenance of chronic inflammation [33].

**Figure 2:**
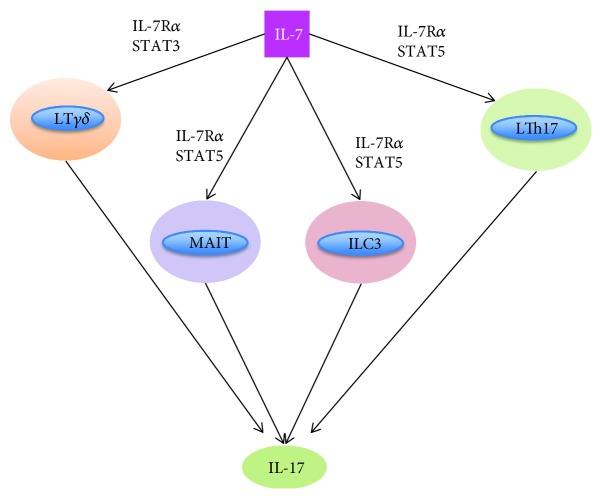
IL-7 stimulates T helper lymphocytes 17 (LTh17), innate immune cells like *γδ* LT, mucosa-associated invariant T (MAIT) cells, and ILC3 to produce IL-17 through activating STAT.

It has recently been shown that IL-7 stimulates not only T helper lymphocytes 17 (LTh17) [34] but also innate immune cells like *γδ* LT [35] and mucosa-associated invariant T (MAIT) cells [36] to produce proinflammatory cytokines, including IL-17 (Figure 2). An interesting fact is that these innate-like T cells (T *γδ*, MAIT and ILC3) are the main source of IL-17A, not Th17 cells, confirming the role of innate immunity as a driver of pathophysiology in SpA [27, 37].

## 2. The Role of IL-7 in Spondyloarthritis-Associated Fibrosis

**Figure 3:**
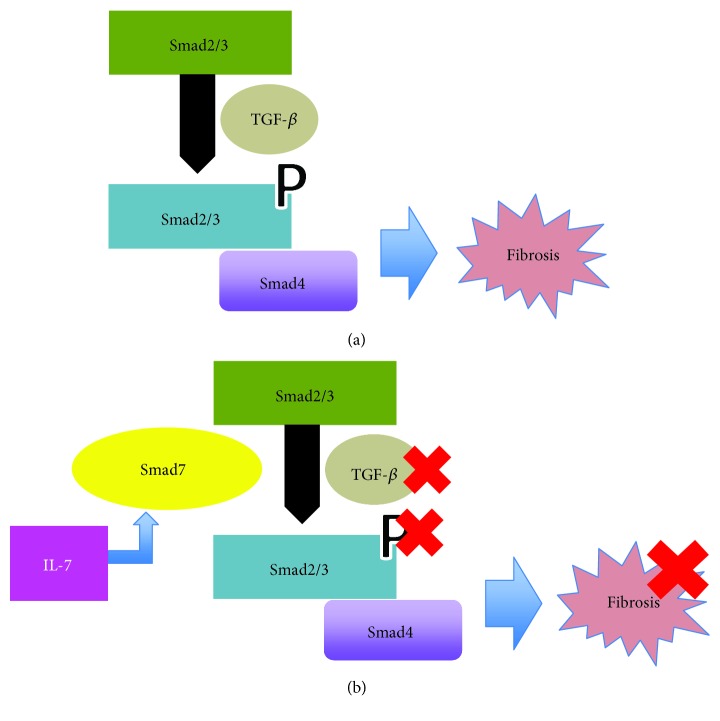
(a) The TGF-*β* receptor phosphorylates Smad2/3. Phosphorylated, they bind to Smad4, and the resulting complex translocates to the nucleus and activates transcription through binding to the CAGA sequence, i.e., initiates signal transduction. (b) Smad7 inhibits Smad2/3 TGF-*β*-mediated phosphorylation and competes with the Smad2/3 binding to the TGF-*β* receptor. In turn, TGF-*β* then induces the production of both IL-7 (b) and Smad7, and this in turn is also stimulated by IL-7, ratifying a negative feedback loop of control of TGF-*β*.

IL-7 counterregulates TGF-driven fibrotic processes and may thus modulate the balance between inflammation and tissue remodeling in SpA [38]. The balance between TGF and IL-7 related to fibrosis is mediated by Smad, and in Figure 3, we elucidate these possible interactions [39–42].

The major thought would be that by blocking IL-7 in patients with SpA in an early period of inflammation, we could prevent the process of tissue repair/fibrosis that is the result of sustained inflammation. In theory, TGF antagonizes IL-7 in a negative feedback process. By inhibiting IL-7, and consequently inflammation, we may be able to prevent fibrosis. Studies to elucidate this mechanism between IL-7 × TGF × inflammation/fibrosis need to be performed.

To resume, TGF-*β* and IL-7 share a reciprocal relationship of antagonism, each of which is capable of downregulation. Indeed, the ability of TGF-*β* to inhibit IL-7-induced pre-B cell proliferation was recognized shortly after identification of IL-7. While the mechanisms and implications of this antagonistic correlation are not yet well clarify, the potential role of these two molecules in several cell populations of immunity suggests that this interaction has important influence on immunological regulation [2, 43].

## 3. IL-7 and Correlation between SpA and MAIT Cells

MAIT cells can act on both the innate and the adaptive immune systems [37]. Because they are unconventional T cells, they produce cytokines faster than conventional T cells and may have both Th1 profile (tumor necrosis factor (TNF) and interferon-gamma (IFN-*γ*)) and a Th17 response (IL-17A) [44]. These cells appear in large numbers in humans, accounting for 1 to 10% of circulating T cells, 20 to 45% of T cells in the liver, and 3 to 5% of lymphoid cells in the intestinal mucosa [27].

MAIT cells emigrate from the thymus and mature in the intestine. This process of maturation in the epithelial cells of the gut therefore depends on local microbial flora as well as B cells [27]. Since alteration in the composition of the microbiota has been associated with the development of several inflammatory arthritis [45], this type of interaction with intestinal microbioma makes MAIT cells a very interesting cell for understanding the pathogenesis of SpAs.

Therefore, high levels of IL-7 have been demonstrated both in the intestinal tissue and in the inflamed joint tissue of patients with AS [32, 46]. Elevated levels of IL-17 have been attributed to the relationship of MAIT cells and IL-7, and this phenomenon may extend to Th17 cells, since Th17 cells also have IL-7 receptor (IL-7R), which may be associated with susceptibility to SpA [27].

MAIT cells although are distinct in their development and have MHC restriction when compared to Th17 cells represent an abundant and highly conserved semi-invariant T cell population that produces IL-17, a major proinflammatory cytokine thought to be involved in SpA pathogenesis.

## 4. The Role of IL-7 into SpA through LT*γδ*

Approximately 30 years ago *γδ* T cells were discovered [47, 48], and since then, *γδ* T cells have been associated with different infections and tumors, as well as autoimmune diseases, like SpA in humans [49, 50]. IL-7 has therefore been described as an essential cytokine in the regulation of development and homeostasis for *γδ* T cells [51, 52].

The first association between T cells and production of IL-17/IL-22 in human SpA has been described by Kenna et al. [53]. Corroborating this finding, analyses of tissue samples from patients with enthesitis-related arthritis [54], reactive arthritis or undifferentiated SpA [55], and juvenile idiopathic arthritis (JIA) patients [56] showed increased IL-17 levels produced by *γδ* T cells in both blood and synovial fluid.

IL-17-producing *γδ* cells may not depend on STAT3 [57], but they are rapidly responsive to IL-23 signaling via STAT3 [58]. Parallel to this, Michel et al. [35] showed a study that identified the ability of IL-7 to activate STAT3 and stimulate IL-17 production. In this way, we can infer that IL-7 is capable of stimulating the production of inflammatory cytokines such as IL-17 by *γδ* T cells and therefore link to the pathophysiology of SpA.

However, another study demonstrated that the maintenance of Th17 cells via the T cell receptor (TCR*αβ*) by IL-7 is mediated by STAT5 [59], which seems paradoxical since STAT5 can antagonize Th17 differentiation. Further studies are needed to better characterize these interactions.

## 5. The Role of IL-7 into Th17 Cells

Th17 cells were discovered in 2005 [60] and appear to coordinate the body's defense against extracellular bacteria and fungi in some specific sites such as the gastrointestinal barrier, respiratory tract, and skin [61]. Th17 cells have the potential to interconnect innate and adaptive immunity, and associated chemokines induce the attraction of other types of Th cells at the sites of infection [62–64]. It has been implicated in the pathogenesis of several immunomediated inflammatory diseases, such as encephalomyelitis, inflammatory bowel disease, systemic lupus erythematosus (SLE), Sjögren's syndrome, rheumatoid arthritis, and SpA [65–69].

The role of Th17 cells is well defined in the development of SpA, and the use of the therapy either by blocking the polarization of Th naive to Th17 [70] or by directly inhibiting the IL-17 cytokine [71] is already performed in a clinical practice.

According to the results of the study by Liu et al., IL-23 promotes via STAT3 the differentiation of Th17, while IL-7 is crucial for the survival and expansion of Th17 through STAT5 signaling, which cannot be blocked by IL-23p19 specific antibody [59]. It also appears that there is a connection between the IL-7 and IL-23 pathways, since the requirement for the IL-23 receptor is required in the reexpression of IL-7R*α* in effector and Th17 memory cells [72]. These findings suggest that IL-23 and IL-7 have roles in the development of Th17 but perhaps in distinct phases.

## 6. IL-7 and the Correlation between Enthesitis and ILC3

Another group of cells that integrate innate immunity are innate lymphoid cells (ILCs) [73, 74]. ILCs are cells that constitute mucosal tissues and demonstrate the characteristic of rapid response to infection by pathogens or to tissue damage [75, 76]. Three groups of ILCs are now recognized based on the properties of cytokines: ILC1 expresses the T-bet transcription factor and produces interferon-*γ* (IFN-*γ*) in addition to mediating immunity against intracellular pathogens and tumors; ILC2 mainly produce IL-5 and IL-13; ILC3s are an important source of cytokines type 17, IL-22 and IL-17 [14].

Intact lymphoid cells from group 3 (ILC3) are able to promote lymphoid organogenesis and potentiate immune responses against fungal and bacterial infection, through the production of IL-17 and IL-22. This type of inflammatory response of the ILC3 is correlated to SpA pathogenesis.

IL-7R*α* signaling that regulates the development and/or maintenance of ILC remains poorly understood [77]. What is already known is that mice with IL-7 deficiency severely reduced the number of all ILC3 populations [78] and therefore exhibits defective lymph node development [79].

Parallel to this, Ciccia et al. [14] confirmed that IL-7-expressing IL-7-specific epithelial cells stimulate ILC3 differentiation, thus increasing IL-17 and IL-22 expression. This study ratifies the suggestion of a fundamental role of these specialized epithelial cells in the activation and amplification of the innate intestinal immune response in patients with AS resulting in active ILC3 differentiation.

Recently, a subset of T cells in the enthesis, highly responsive to IL-23, has been described in a mouse model of SpA [80]. Although the intestinal presence of these cells has not been studied in the work of Sherlock et al. [80], several immunological similarities are shared between murine entheseal T cells and ILC3 that were described in the study by Ciccia et al. [14]. Both murine and human cells were in fact lyn negative, express IL-23R, and produce IL-17 and IL-22. With these findings, it can be assumed that perhaps innate immunity cells, such as ILC3, are more prominently associated with enthesitis/SpA than conventional T cells, and therefore as the presence of IL-7R in those cells is a fact, the IL-7 role could be presumed.

## 7. Conclusion

SpA has an important pathophysiological component of the type 17 signature with production of proinflammatory cytokines such as IL-17. IL-7 is a cytokine correlated with cells of innate immunity (ILC, MAIT, and LT*γδ*) and adaptive (Th17 cells) and seems to play an important role in the STAT transcriptional stimulus in the type 17 response, either in the production of cytokines or in the survival and expansion of IL-17-producing cells.

IL-7 appears to be more important than IL-23 in the polarization of the type 17 signature, since the IL-7 receptor is present in the key cells of innate immunity responsible for the polarization of type 3 response. It is an interesting target for the development of research including in the context of therapy trials for SpA.
